# Low prevalence but high resistance of Extended Spectrum Beta-lactamase producing *Klebsiella pneumoniae* isolated from Hospitals in the Ashanti Region of Ghana

**DOI:** 10.4314/ahs.v25i2.4

**Published:** 2025-06

**Authors:** Elizabeth Akua Baidoo, Vivian Etsiapa Boamah, Hayford Odoi, Yaw Duah Boakye, Christian Agyare

**Affiliations:** 1 Microbiology Section, Department of Pharmaceutics, Faculty of Pharmacy and Pharmaceutical Sciences, Kwame Nkrumah University of Science and Technology, Kumasi, Ghana; 2 Department of Pharmaceutical Microbiology, School of Pharmacy, University of Health and Allied Sciences, Ho, Ghana

**Keywords:** ESBLs, *K. pneumoniae*, antibiotic resistance, Ghana

## Abstract

**Background:**

Antibiotic resistance continues to be a threat to public health worldwide, with its associated increased healthcare costs and loss of human lives. Despite the increasing reports of multi-drug resistant (MDR) *Klebsiella pneumoniae* (*K. pneumoniae*), its prevalence and resistance patterns remain under-reported in several developing countries, including Ghana.

**Objectives:**

This study hence sought to investigate the prevalence and resistance patterns of *K. pneumoniae* isolates from five selected hospitals in the Ashanti region of Ghana.

**Methods:**

*K. pneumoniae* isolates were identified using biochemical tests, API 20 E and Matrix Assisted Laser Desorption Ionisation-Time of Flight Mass Spectrometry (MALDI-TOF MS). AST and ESBL production were determined phenotypically and via PCR

**Results:**

Twenty (20) *K. pneumoniae* isolates were confirmed from the samples. The isolates were resistant to ampicillin (100%), ciprofloxacin (76.67%), co-trimoxazole (75%), chloramphenicol (63.33%) and cephalosporins (76.67 to 55%). Eighteen of the K. pneumoniae isolates (90%) were multi-drug resistant (MDR) while 60% of the isolates (n=12) produced Extended Spectrum Beta-Lactamases (ESBL) with blaCTX-M, blaSHV and blaTEM β-lactamase resistant encoding genes confirmed in 83, 75 and 33% of the isolates respectively.

**Conclusion:**

There is low prevalence but high multidrug resistance and high production of ESBL among *K. pneumoniae* isolates from the selected hospitals.

## Introduction

Antimicrobial resistance (AMR) presents a serious worldwide risk of increasing health concern due to the spread and emergence of multidrug-resistant (MDR) microbes^1^,^2^. AMR is usually caused by the excessive use of antibiotics in both clinical and non-clinical settings, unregulated supply of antimicrobials, poor drug quality^3^,^4^, non-compliance to antibiotic therapy, over-the-counter sale of antibiotics, poor sanitation and the release of antimicrobials into the environment^5^,^6^. These factors result in increased chances of the acquisition of resistance by microorganisms through selective pressure^7^.

*Klebsiella pneumoniae* (*K. pneumoniae*) is an encapsulated, non-motile Gram-negative commensal^8^ having a wide range of virulence factors that assist the bacterium in infection and antimicrobial resistance^9^. *K pneumoniae* is a significant cause of nosocomial infections especially within units of hospitals such as intensive care units and neonatal units where there is excessive use of antibiotics^10^,^11^.

The microbe is currently resistant to a wide range of broad-spectrum antibiotics including beta-lactam antibiotics, aminoglycosides and fluoroquinolones 12. Of prime concern is the resistance of *K pneumoniae* to cephalosporins and carbapenems as a result of the production of extended-spectrum beta-lactamases (ESBLs)^13^. Infections caused by microorganisms producing extended spectrum of β-lactamases (ESBL), notably *Klebsiella pneumoniae*, are escalating^14^ in both clinical settings and community settings^15^.

Multidrug resistance in *K pneumoniae* has been reported globally in recent times however, very few of the reports have been from developing countries, including Ghana^16^,^17^. Hence, this study aimed to investigate the prevalence, and resistance patterns and to identify beta-lactamase resistance genes within *K. pneumoniae* isolates from selected hospitals in the Ashanti region of Ghana.

## Methods

### Study sites

The study was conducted in five hospitals in the Ashanti Region of Ghana including Komfo Anokye Teaching Hospital, Manhyia Government Hospital, Suntreso Government Hospital, KNUST Hospital all located in the Kumasi Metropolis, and Agogo Presbyterian Hospital located in the Asante-Akyem Central Municipal from May 2017 to November 2018. All selected hospitals are secondary-level healthcare facilities except for Komfo Anokye Teaching Hospital (KATH) which is a tertiary-level healthcare facility. These hospitals provide healthcare services to the majority of the people in the Kumasi Metropolis and Asante-Akyem Central Municipal, respectively.

### Isolation of *Klebsiella pneumoniae* strains

Urine, stool, wound, and nasal samples were collected from patients reporting to the laboratory for analysis. The collected samples were given codes. The samples were collected into sterile containers and transported to the Microbiology laboratory, Department of Pharmaceutics, Kwame Nkrumah University of Science and Technology, Kumasi, on ice. The samples were inoculated into nutrient broth (Oxoid, Basingstoke, UK), incubated at 37°C for 24 h, and then stored in the refrigerator. The samples were cultivated in nutrient broth and MacConkey broth (Oxoid, Basingstoke, UK) supplemented with vancomycin at 37°C for 24 h. A loopful from the test tubes with growth were subsequently streaked on MacConkey agar (Oxoid, Basingstoke, UK) and blood agar. Single lactose fermenting colonies were fished and inoculated into glycerol broth and incubated at 37°C for 24 h. Biochemical tests including Sulphur indole motility (SIM) test, methyl red test, citrate test, and Triple sugar iron (TSI) test (Oxoid, Basingstoke, UK) were performed to aid in the identification of the isolates. The identities of the isolates after the biochemical tests were confirmed using API 20E (bioMerieux Inc, Durham, USA) and MALDI-TOF MS (Bruker Daltonics, Bremen, Germany).

### Antimicrobial susceptibility testing

Antimicrobial susceptibility testing was performed according to the Kirby Bauer Disk Diffusion Assay method using breakpoint values from^18^. Antibiotics which were tested against the *K. pneumoniae* isolates are Ampicillin (10 µg), Cefuroxime (30 µg), Chloramphenicol (30 µg), Co-trimoxazole (25 µg), Gentamicin (30 µg), Ciprofloxacin (5 µg), Levofloxacin (5 µg), Amikacin (30 µg), Ceftriaxone (30 µg), Cefpodoxime (10 µg), Ceftazidime (10 µg), Aztreonam (30 µg) all from Oxoid, Basingstoke, UK as test antibiotics. A revived culture of the *K pneumoniae* isolate plated on nutrient agar (Oxoid, Basingstoke, UK) was inoculated into sterile saline and mixed uniformly to obtain 1.5 x 108cfu/ml standardized inoculum. A sterile cotton swab was dipped into the suspension and spread evenly on the agar plate by swabbing in three different directions. Antibiotic disks were dispensed unto the inoculated agar plate using an Oxoid disk dispenser (Oxoid, Basingstoke, UK). It was left on the bench for 15 minutes and then incubated at 37°C for 20 hours. *Klebsiella pneumoniae* (clinical strain) was used as a positive control 19.

### Double disk synergy test (DDST) for detection of ESBL-producing *K. pneumoniae*

The DDST is used for the detection of beta-lactamases that are inhibited by beta-lactamase inhibitors such as clavulanic acid. This was done by dispensing the antibiotics disks cefpodoxime (10 µg), ceftazidime (10 µg) and ceftriaxone (30 µg) (Oxoid, Basingstoke, UK) on Mueller-Hinton agar (Oxoid, Basingstoke, UK) inoculated with a 0.5 McFarland standardized overnight culture. The plates were then incubated at 37°C for 24 h. Isolates with zones of inhibition 21 mm for cefpodoxime (10 µg), 22 mm for ceftazidime (10 µg) and 23 mm for ceftriaxone (30 µg) after incubation at 37°C for 24 h were suspected to be ESBL producers^20^. Isolates that tested positive for the ESBL screening were passed through the Disc synergy test for confirmation. The combination disc, ceftazidime (30 µg) plus clavulanic acid (10 µg) and ceftazidime (30 µg) only were introduced onto plates of Mueller-Hinton agar inoculated with the *K pneumoniae* isolates (0.5 McFarland standard). The plates were incubated at 37°C for 20 h. After incubation, the zones were read as the diameter from one zone edge to the other^19^. A difference of ≥ 5 mm in the zone of growth inhibition of the two discs indicated the presence of an ESBL-producing bacteria.

### Detection of blaCTX-M, blaSHV and blaTEM ESBL encoding genes in ESBL-producing *K. pneumoniae* isolates

The DNA of the *K. pneumoniae* isolates were extracted using a modified form of the method described by^21^. The ESBL gene blaCTX-M, was detected using a method described by^22^ whereas blaSHV and blaTEM were detected as described by^23^. A reaction mixture of volume 25 µL consisting of 16.8 µL of nuclease-free water (ThermoFisher Scientific Limited, Karlsruhe, Germany), 2.5 µL of 10X buffer, 2 µL of 0.5mM MgCl2, 0.5 µL of dNTP, and 0.5 µL each of 10 µM forward and reverse primer (Table 1), 0.125 µL of Taq and 2 µL of the DNA template were used. The cycling conditions for blaCTX-M were denaturation at 94°C for 3 min, followed by 35 cycles of denaturation at 94°C for 45 s, annealing at 62oC for 45 s, elongation at 72°C for 30 s, and then final elongation at 72°C for 10 min. For blaSHV and blaTEM, there was denaturation at 94°C for 3 min, followed by 35 cycles of denaturation at 94°C for 45 s, annealing at 56°C for 35 s, elongation at 72°C for 30 s, and then finally elongated at 72°C for 10 min. The amplicons (10 µL) were stained with bromothymol blue (New England Biolabs, Massachusetts, USA) and further loaded unto 1.5% ethidium bromide-stained agarose gel (Sigma-Aldrich Scientific, Copenhagen, Denmark). The agarose gel was subjected to electrophoresis at 100V for 45 min. The bands were visualized with the aid of a transilluminator (Fotodyne, Hartland, WI, USA).

**Table 1:** Primer Pairs Used in Detecting ESBL Genes

Gene	Primer	Sequence (5″ – 3″)	Size	Reference/Supplier/Place
***bla* TEM**	TEM – F	ATGAGTATTCAACATTTCCG	861 bp	24
	TEM – R	CCAATGCTTAATCAGTGAGC		IDT, California, USA
***bla* SHV**	SHV-F	CTTTACTCGCTTTATCG	827 bp	24
	SHV-R	TCCCGCAGATAAATCACCA		IDT, California, USA
***bla* CTX-M**	CTX-M-F	TTTGCGATGTGCAGTACCAGTAA	544 bp	25
	CTX-M-R	CGATATCGTTGGTGGTGCCATA		IDT, California, USA

### Statistical Analysis

Descriptive analysis such as arithmetic means, frequencies, and percentages were determined using SPSS (IBM, USA, version 20.0) and Microsoft Excel (Microsoft Office Professional Plus 2013, USA)

## Results

### Prevalence and Antibiogram profiles of *Klebsiella pneumoniae* isolates

**Figure 1:**
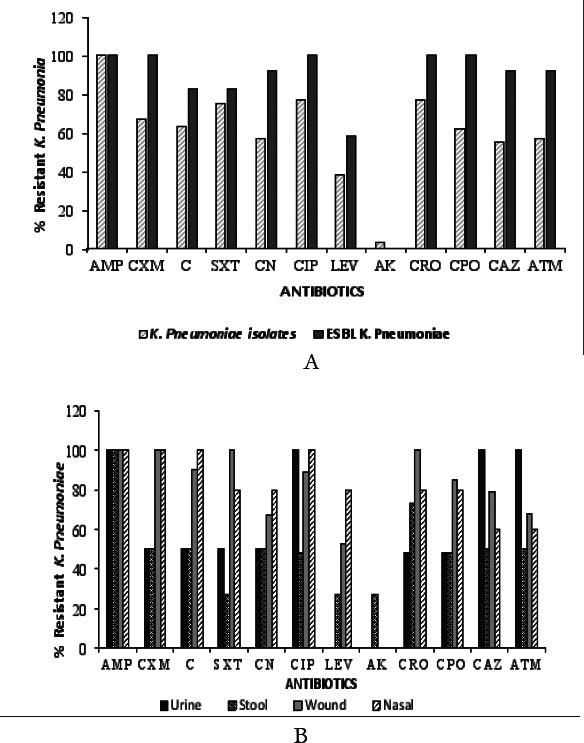
A: Sensitivity pattern of *K. pneumoniae* Isolates and ESBL producing *Klebsiella pneumoniae* against Selected Antibiotics. B: Sensitivity pattern of *K. pneumoniae* Isolates among the various samples. AMP: Ampicillin (10 µg); CXM: Cefuroxime (30 µg); C: *Chloramphenicol* (30 µg); SXT: *Co-trimoxazole* (25 µg); CN: *Gentamicin* (30 µg); CIP: *Ciprofloxacin* (5 µg); LEV: *Levofloxacin* (5 µg); AK *Amikacin* (30 µg); CRO: *Ceftriaxone* (30 µg); CPO: *Cefpodoxime* (10 µg); CAZ: Ceftazidime (10 µg); ATM: Aztreonam (30 µg)

A total of 20 *K. pneumoniae* isolates were obtained from 510 clinical samples obtained from the selected hospitals (196 urine, 77 stool, 130 wound swabs, and 107 nasal swab samples). *K. pneumoniae* isolates were obtained from wound samples (n=9, 45%), nasal swabs (n=5, 25%), stool samples (n=4, 20%), and urine samples (n= 2, 10%). Resistance of *K pneumoniae* to all the 12 antibiotics tested was in the range of 0% to 100% (Figure 1). The isolates were resistant to ampicillin (100%), ciprofloxacin (77%), co-trimoxazole (75%), and the cephalosporins [ceftriaxone (77%), cefuroxime (67%), cefpodoxime (62%), and ceftazidime (55%)]. The least resistance occurred with amikacin (3%) among all the samples collected.

Eighteen (90%) of the isolates were multi-drug resistant (MDR) with 25% of these isolates being resistant to 11 out of the 12 selected antibiotics (Table 2).

**Table 2:** Antibiotic resistance pattern of *K. pneumoniae* isolates and prevalence of ESBL encoding genes in clinical samples from the various hospitals

Antibiotics isolates are resistant towards	KATH (n=12)					Manhyia (n=1)				Suntreso (n=4)					Agogo (3)		
	n	U	S	W	N	U	S	W	N	U	S	W	N	U	S	W	N
**Number of samples**	**5** **1** **0**	**3** **3**	**1** **3**	**45**	**71**	**3** **2**	**2** **2**	**8**	**7**	**3** **0**	**20**	**30**	**1** **5**	**7**	**2** **1**	**2** **7**	**2** **4**
**Number of isolates**	**2** **0**	**1**	**2**	**4**	**5**	**0**	**1**	**0**	**0**	**1**	**1**	**2**	**0**	**0**	**0**	**3**	**0**

**AMP-AK**	1	0	0	0	0	0	0	0	0	1	0	0	0	0	0	0	**0**
**AMP-CIP**	1	0	0	0	0	0	0	0	0	0	1	0	0	0	0	0	**0**
**AMP-C-CRO**	1	0	1	0	0	0	0	0	0	0	0	0	0	0	0	0	**0**
**AMP-CXM-C-SXT-CIP-CRO**	1	0	0	0	0	0	0	0	0	0	1	0	0	0	0	0	**0**
**AMP-CXM-C-SXT-CN-CIP**	1	0	0	0	1	0	0	0	0	0	0	0	0	0	0	0	**0**
**AM P-CXM-CN-CIP-CRO-CPO**	1	0	1*^	0	0	0	0	0	0	0	0	0	0	0	0	0	**0**
**AMP-CXM-C-SXT-LEV-CRO-CPO-CAZ**	1	0	1	0	0	0	0	0	0	0	0	0	0	0	0	0	**0**
**AMP-CXM-C-SXT-CIP-LEV-CRO-CPO**	1	0	0	0	1	0	0	0	0	0	0	0	0	0	0	0	**0**
**AMP-CXM-C-SXT-CN-CIP-CRO-CPO-CAZ**	1	1*^	0	0	0	0	0	0	0	0	0	0	0	0	0	0	**0**
**AMP-CXM-C-SXT-CN-CIP-LEV-CRO-CPO**	1	0	0	0	0	0	0	0	0	0	0	0	0	0	0	1	**0**
**AMP-CXM-C-SXT-CIP-CRO-CPO-CAZ-ATM**	1		0	0	0	0	0	0	0	0	0	0	0	0	0	1*^^#^	**0**
**AMP-CXM-C-SXT-CN-CIP-CRO-CPO-CAZ-ATM**	2	0	0	2*	0	0	0	0	0	0	0	0	0	0	0	0	**0**
**AMP-CXM-C-CN-CIP-LEV-CRO-CPO-CAZ-ATM**	1	0	0	0	0	0	0	0	0	0	0	0	0	0	0	1*	**0**
**AMP-CXM-SXT-CN-CIP-LEV-CRO-CPO-CAZ-ATM**	1	0	0	0	1*^^#^	0	0	0	0	0	0	0	0	0	0	0	**0**
**AMP-CXM-C-SXT-CN-CIP-LEV-CRO-CPO-CAZ-ATM**	5	0	0	1*^	2*^′*^^#^	0	1*^	0	0	0	0	1*^^#^	0	0	0	0	**0**

**ESBL gene amplification**																	
**CTX-M**	**1** **0**	1	1	2	3	0	0	0	0	0	0	1	0	0	0	2	**0**
**SHV**	**9**	1	1	1	3	0	1	0	0	0	0	1	0	0	0	1	**0**
**TEM**	**4**	0	0	0	2	0	0	0	0	0	0	1	0	0	0	1	**0**

**Key:** AMP: Ampicillin (10 µg); CXM: Cefuroxime (30 µg); C: Chloramphenicol (30 µg); SXT: Co-trimoxazole (25 µg); CN: Gentamicin (30 µg); CIP: Ciprofloxacin (5 µg); LEV: Levofloxacin (5 µg); AK: Amikacin (30 µg); CRO: Ceftriaxone (30 µg); CPO: Cefpodoxime (10 µg); CAZ: Ceftazidime (10 µg); ATM: Aztreonam (30 µg) n: Total number of isolates; U: Urine; S: Stool; W Wound; N: Nasal

* CTX-M gene

^ SHV gene

# TEM gene

After ESBL screening and confirmation, 12 (60%) of the *K. pneumoniae* isolates were found to be ESBL producers. Nine (9) of the ESBL producers were obtained from KATH, two (2) were from Agogo Presbyterian Hospital, and one (1) from Suntreso and Manhyia Government Hospitals each (Table 2). The ESBL producers were highly resistant to all the antibiotics except for amikacin.

### Detection of ESBL blaCTX-M, blaSHV and blaTEM resistance encoding genes in *K. pneumonaie* isolates

**Figure 2:**
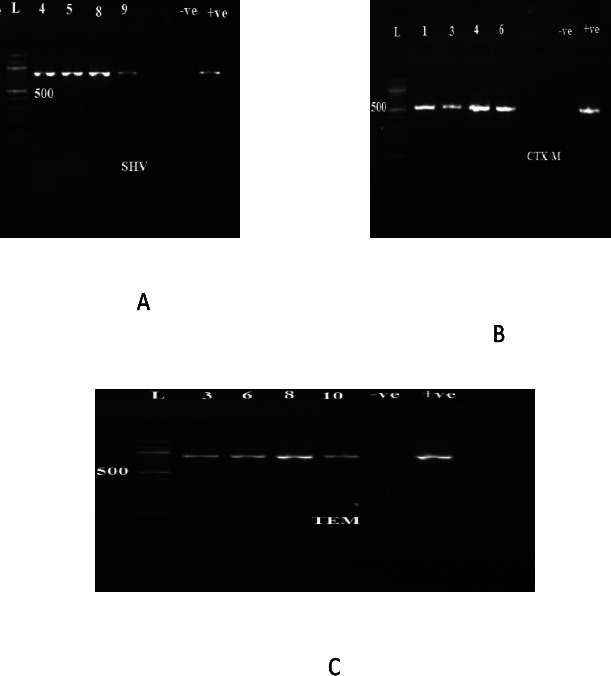
An electrophoretic gel image showing the 827 bp PCR amplicon of blaSHV (A), 544bp PCR amplicon of blaCTX-M (B) and 861bp PCR amplicon of blaTEM (C) in ESBL producing *K pneumoniae*. Key: L: 100 bp DNA ladder; -ve: negative control; +ve: positive control

The ESBL resistance encoding genes blaCTX-M (n=10, 83%), blaSHV (n=9, 75%) and blaTEM (n=4, 33%) were detected in the ESBL producers (Figure 2). blaCTX-M was the most prevalent of the three genes. Only one of the ESBL-producing *K. pnemoniae* isolates did not carry any of the three genes. Three of the ESBL-producing *K pneumoniae* isolates haboured all three genes. Four of the ESBL-producing *K. pneumoniae* isolates had two of the genes and only one gene was found in four of the ES-BL-producing *K pneumoniae* isolates (Table 2).

## Discussion

In recent years, there have been increasing reports of multidrug-resistant infections caused by *K. pneumoniae* 26. This is regarded as a concerning issue in both developed and developing nations as antibiotics are used so widely in medicine^27^. *K. pneumoniae* is a common bacterial pathogen implicated in nosocomial infections globally. This microorganism may be identified in wound infections, systemic bacteraemia, urinary tract infections as well as upper respiratory tract infections^28^. This study reports the prevalence, resistance patterns and ESBL resistance mechanisms of *K. pneumoniae* in selected health facilities in Ashanti Region, Ghana.

The prevalence of *K. pneumoniae* in this present study was 3.99%. Most of the isolates were acquired from wound swabs (45%) while urine specimens haboured the least (10%) isolates. A similar study carried out in Iran reported a prevalence of 10.2% with the largest number of isolates being obtained from urine and the least from blood^29^. This variation may be a result of differences in the demographics of the subjects involved in the study.

The *K. pneumoniae* isolates demonstrated high resistance to the reference antibiotics used. All the isolates were resistant to ampicillin (100%). Resistance to ampicillin is said to be an inherent feature of *Klebsiella* spp. resulting in the production of chromosomally inscribed beta-lactamase gene SHV-1 by members of the genus^30^.

Third-generation cephalosporins are commonly used in the treatment of *K. pneumoniae* infections because of their broad spectrum of activity and their lesser side effects 31. The isolates were highly resistant to the cephalosporins used in this study. Sixty-five percent of the isolates were resistant to all third-generation cephalosporins used. This finding is similar to that of^32^, who indicated that 63.4% of their *K. pneumoniae* isolates were resistant to second and third-generation cephalosporins.

Resistance to the quinolones used in this study, especially ciprofloxacin was high (76.67%). This resistance rate is higher than that of Apondi et al. (2016) who reported a ciprofloxacin-resistance rate of 44%. However, this finding was similar to that of^33^, who reported a ciprofloxacin resistance rate of 69.4%. Ciprofloxacin resistance (77%) was higher than levofloxacin resistance (38.33%). This disparity is similar to the findings of^34^, who reported a levofloxacin resistance rate of 33.3%. This difference in resistance rate may result from the more frequent use of ciprofloxacin as compared to levofloxacin in Ghana. Ciprofloxacin is recommended for the empirical treatment of uncomplicated urinary tract infections in Ghana^35^.

Aminoglycoside resistance was relatively low: amikacin had the least resistance (3.33%) and gentamicin had a resistance rate of 56.67%.^36^ Reported resistance rates of 4.16% and 16.05% for amikacin and gentamicin respectively in a similar study. The elevated susceptibility of the *K. pneumoniae* to aminoglycosides may be ascribed to a decrease in the use of aminoglycosides following the introduction of third-generation cephalosporins and fluoroquinolones with less serious side effects and a wider spectrum of activity^37^.

Resistance to co-trimoxazole (75%) and chloramphenicol (63%) was high. High resistance rates to these antibiotics i.e co-trimoxazole (73%) and chloramphenicol (75%) have been reported in a nationwide study carried out in Ghana by^38^. These high resistance rates may be attributed to the easy accessibility of these antibiotics to the general public and their misuse.

The emergence of multidrug-resistant bacteria is becoming a global battle as it is linked to high mortality, prolonged hospital stay, reduced effectiveness of drugs, and high medical costs^39^. Acquiring plasmids with multiple antimicrobial resistance genes is usually the cause of antimicrobial resistance in *K. pneumoniae*^40^. Ninety percent of the *K. pneumoniae* isolates were found to be multidrug-resistant. A similar study revealed that 94.7% of *K. pneumonaie* isolates from clinical samples were multidrug-resistant^41^. This was further confirmed by the findings of 33, who also reported a high incidence of multidrug resistant *K. pneumoniae* isolates.

ESBLs which are plasmid-mediated enzymes that confer resistance to penicillins, cephalosporins and monobactams are commonly found in *K. pneumoniae* 42. This study reports a high prevalence of ESBLs (60%) among isolates. This finding corroborates that of^43^ who reported a 61.9% prevalence of ESBL producers among *K. pneumoniae* isolates in Accra as well as that of^44^ who reported an ESBL prevalence of 57.8% among *K. pneumoniae* isolates in Kumasi. The ESBL-producing *K pneumoniae* were highly resistant to most of the antibiotics except for amikacin. This may be due to the cross-resistance conferred by the ESBL genes to the isolates^45^. Molecular identification of ESBL-carrying isolates is crucial in determining the origin of these pathogens, and in supplying pertinent data about the distribution of clonally related or unrelated ESBL genotypes^42^. There was a high prevalence of ESBL resistance encoding genes, blaCTX-M, blaSHV and bla TEM among the ESBL producing isolates. blaCTX-M (83%) was the most prevalent of the three genes followed by blaSHV (75%) and then blaTEM (33%). blaCTX-M form of extended-spectrum β-lactamases has become predominant in many parts of the world^46^. In a related study, blaSHV-11, blaCTX-M-15, and blaTEM-1 encoding genes were similarly identified in *Klebsiella pneumoniae* isolates from Iran^47^. Only one phenotypic ESBL producing *K. pneumoniae* isolates harboured none of the researched ESBL genes (CTX-M, SHV, and TEM). This isolate may however possess an AmpC gene that has not been considered in the present research.

The high rate of resistance of the *K. pneumoniae* isolates may be attributed to the misuse of antibiotics by healthcare professionals and patients through practices such as over-prescription, non-adherence to the prescribed antibiotic courses by patients and the indiscriminate sale of antibiotics^48^. The non-human use of antibiotics such as in animal husbandry and food preservation is also an important cause of antimicrobial resistance^49^. The incidences of the use of substandard antibiotics with a lower amount of the antibiotic than is required is also on the rise^50^. These call for strict measures to monitor anregulate the use of antibiotics in both clinical and community environments.

## Conclusion

A low prevalence (20, 4%) of *K. pneumoniae* isolates were obtained from all the clinical samples examined during the study. The *K. pneumoniae* isolates were highly resistant to most of the antibiotics except amikacin. A high proportion of the isolates were multidrug-resistant (90%) and ESBL producers (60%). blaCTX-M (83%), blaSHV (75%) and blaTEM (33%) ESBL resistance encoding genes are present in the ESBL producing *K. pneumoniae* isolates from the Ashanti region of Ghana.

## Ethical considerations

This study was approved by the Committee on Human Research Publications and Ethics of the School of Medicine and Dentistry, Kwame Nkrumah University of Science and Technology, Kumasi and the Komfo Anokye Teaching Hospital, Kumasi with the reference number CHPRE/RC/032/18. The permission of the authorities of the various hospitals and patients were sought before the collection of the samples was done.

## Abbreviations

ESBL: Extended Spectrum Beta-Lactamase
EUCAST: European Committee on Antimicrobial Susceptibility Testing
KATH: Komfo Anokye Teaching Hospital
KNUST: Kwame Nkrumah University of Science and Technology
MALDI-TOF: Matrix Assisted Laser Desorption Ionization – Time of Flight
MS: Mass Spectrometry
PCR: Polymerase Chain Reaction

## References

[1] Aslam B, Wang W, Arshad MI, Khurshid M, Muzammil S, Rasool MH (2018). Antibiotic resistance: a rundown of a global crisis. Infect Drug Resist.

[2] Prestinaci F, Pezzotti P, Pantosti A (2015). Antimicrobial resistance: a global multifaceted phenomenon. Pathog Glob Health.

[3] Byarugaba DK (2004). Antimicrobial resistance in developing countries and responsible risk factors. Int J Antimicrob Agents.

[4] Okeke IN, Klugman KP, Bhutta ZA, Duse AG, Jenkins P, O'Brien TF (2005). Antimicrobial resistance in developing countries. Part II: strategies for containment. Lancet Infect Dis.

[5] Okeke IN, Laxminarayan R, Bhutta ZA, Duse AG, Jenkins P, O'Brien TF (2005). Antimicrobial resistance in developing countries. Part I: recent trends and current status. Lancet Infect Dis.

[6] Ayukekbong JA, Ntemgwa M, Atabe AN (2017). The threat of antimicrobial resistance in developing countries : causes and control strategies. Antimicrob Resist Infect Control.

[7] Michael CA, Dominey-Howes D, Labbate M (2014). The Antimicrobial Resistance Crisis: Causes, Consequences, and Management. Front Public Health.

[8] Ullmann U, Podschunn R (1998). Klebsiella spp. as Nosocomial Pathogens : Epidemiology, Taxonomy, Typing Methods, and Pathogenicity Factors. Clincal Microbiology reviews.

[9] Paczosa MK (2016). Klebsiella pneumoniae : Going on the Offense with a Strong Defense. Microbiology and Molecular Biology Reviews.

[10] Agaba P, Tumukunde J, Tindimwebwa JVB, Kwizera A (2017). Nosocomial bacterial infections and their antimicrobial susceptibility patterns among patients in Ugandan intensive care units: a cross sectional study. BMC Res Notes.

[11] Apondi OE, Oduor OC, Gye BK, Kipkoech MK (2016). High Prevalence Of Multi-Drug Resistant *Klebsiella pneumoniae* In A Tertiary Teaching Hospital In Western Kenya Department of Child Health and Pediatrics, Moi Teaching and Referral Hospital, Eldoret. Kenya. 2 Department. Afr J Infect Dis.

[12] Navon-venezia S, Kondratyeva K, Carattoli A (2017). Klebsiella pneumoniae : a major worldwide source and shuttle for antibiotic resistance. FEMS Microbiol Rev.

[13] Codjoe FS, Donkor ES (2017). Carbapenem Resistance: A Review. Med Sci (Basel).

[14] Gupta A, Ampofo K, Rubenstein D, Saiman L (2003). Extended Spectrum β Lactamase-producing *Klebsiella pneumoniae* Infections: a Review of the Literature. Journal of Perinatology.

[15] Zhang J, Zhou K, Zheng B, Zhao L, Shen P, Ji J (2016). High Prevalence of ESBL-Producing *Klebsiella pneumoniae* Causing Community-Onset Infections in China. Frontiers in Microbiology.

[16] Comandatore F, Sassera D, Ambretti S, Landini MP, Daffonchio D, Marone P (2013). Draft genome sequences of two multidrug resistant Klebsiella pneumoniae ST258 isolates resistant to colistin. Genome Announc.

[17] Codjoe FS, Donkor ES, Smith TJ, Miller K (2019). Phenotypic and Genotypic Characterization of Carbapenem-Resistant Gram-Negative Bacilli Pathogens from Hospitals in Ghana. Microbial Drug Resistance [Internet].

[18] EUCAST (2019). Breakpoint tables for interpretation of MICs and zone diameters [Internet].

[19] EUCAST (2019). Antimicrobial susceptibility testing EUCAST disk diffusion method.

[20] Hackman HK, Osei-Adjei G, Ameko E, Kutsanedzie F, Gordon A, Laryea E (2013). Phenotypic Determination and Antimicrobial Resistance Profile of Extended Spectrum Beta-lactamases in Escherichia coli and Klebsiella pneumoniae in Accra, Ghana. Journal of Natural Sciences Research [Internet].

[21] Queipo-Ortuño MI, De Dios Colmenero J, Macias M, Bravo MJ, Morata P (2008). Preparation of Bacterial DNA Template by Boiling and Effect of Immunoglobulin G as an Inhibitor in Real-Time PCR for Serum Samples from Patients with Brucellosis. Clinical and Vaccine Immunology.

[22] Garcia D de O, Doi Y, Szabo D, Adams-haduch JM, Vaz MI, Leite D (2008). Multiclonal Outbreak of *Klebsiella pneumoniae* Producing Extended-Spectrum ␤-Lactamase CTX-M-2 and Novel Variant CTX-M-59 in a Neonatal Intensive Care Unit in Brazil. Antimicrob Agents Chemother.

[23] Cekanová L, Kolár M, Chromá M, Sauer P, Sedlácková M, Koukalová D (2009). Prevalence of ESBL-positive bacteria in the community in the Czech Republic. Medical Scientific Monitoring.

[24] Cekanová L, Kolár M, Chromá M, Sauer P, Sedlácková M, Koukalová D (2009). Prevalence of ESBL-positive bacteria in the community in the Czech Republic. Medical Scientific Monitoring.

[25] Garcia D de O, Doi Y, Szabo D, Adams-haduch JM, Vaz MI, Leite D (2008). Multiclonal Outbreak of *Klebsiella pneumoniae* Producing Extended-Spectru m ␤-Lactamase CTX-M-2 and Novel Variant CTX-M-59 in a Neonatal Intensive Care Unit in Brazil. Antimicrobial agents and chemotherapy.

[26] Quansah E, Amoah Barnie P, Omane Acheampong D, Obiri-Yeboah D, Odarkor Mills R, Asmah E (2019). Geographical Distribution of β-Lactam Resistance among *Klebsiella* spp. from Selected Health Facilities in Ghana. Trop Med Infect Dis [Internet].

[27] Naqid IA, Hussein NR, Balatay A, Saeed KA, Ahmed HA, Naqid IA (2020). Antibiotic Susceptibility Patterns of Uropathogens Isolated from Female Patients with Urinary Tract Infection in Duhok Province, Iraq. Jundishapur Journal of Health Sciences.

[28] Janssen H, Janssen I, Cooper P, Kainyah C, Pellio T, Quintel M (2018). Antimicrobial-Resistant Bacteria in Infected Wounds, Ghana, 2014. Emerging Infectious Disease Journal.

[29] Babakhani S, Shokri Derikvand S, Nazer MR, Kazemi MJ (2014). Comparison frequency and Determination antibiotic resistance pattern of *Klebsiella* spp. isolated from Nosocomial infection in Khorramabad Shohadaye Ashayer hospital. Bull Env Pharmacol Life Sci.

[30] Stanley IJ, Kajumbula H, Bazira J, Kansiime C, Rwego B, Asiimwe BB (2018). Multidrug resistance among Escherichia coli and Klebsiella pneumoniae carried in the gut of out-patients from pastoralist communities of Kasese district, Uganda. PLoS One.

[31] Krause KM, Serio AW, Kane TR, Connolly LE (2016). Aminoglycosides: An Overview. Cold Spring Harbor Perspective in Medicine.

[32] MANJULA NG, Shivannavar CT, Math GC (2014). Antibiotic Susceptibility Pattern of ESβL Producing *Klebsiella pneumoniae* Isolated from Urine Samples of Pregnant Women in Karnataka. Journal of Clinical and Diagnostic Research.

[33] Apondi OE, Oduor OC, Gye BK, Kipkoech MK (2016). High Prevalence Of Multi-Drug Resistant Klebsiella Pneumoniae In A Tertiary Teaching Hospital In Western Kenya Department of Child Health and Pediatrics, Moi Teaching and Referral Hospital, Eldoret. Kenya. 2 Department. Afr J Infect Dis.

[34] Huang Y, Ogutu JO, Gu J, Ding F, You Y, Huo Y (2015). Comparative Analysis of Quinolone Resistance in Clinical Isolates of Klebsiella pneumoniae and Escherichia coli from Chinese Children and Adults. Biomed Res Int.

[35] Owusu H, Thekkur P, Ashubwe-Jalemba J, Hedidor GK, Corquaye O, Aggor A (2022). Compliance to Guidelines in Prescribing Empirical Antibiotics for Individuals with Uncomplicated Urinary Tract Infection in a Primary Health Facility of Ghana, 2019-2021. Int J Environ Res Public Health [Internet].

[36] Tang X, Cai S, Chen J, Xu L, Wang J, Li W (2013). Aminoglycoside Resistance in Clinical *Klebsiella pneumoniae* Isolates. J Pure Appl Microbiol.

[37] Krause KM, Serio AW, Kane TR, Connolly LE (2016). Aminoglycosides : An Overview. Cold Spring Harbor Perspective in Medicine.

[38] Newman MJ, Frimpong E, Donkor ES, Opintan JA, Asamoah-Adu A (2011). Resistance to antimicrobial drugs in Ghana. Infect Drug Resist.

[39] Tanwar J, Das S, Fatima Z, Hameed S (2014). Multidrug Resistance: An Emerging Crisis. Interdiscip Perspect Infect Disease.

[40] Salawudeen A, Raji YE, Jibo GG, Desa MNM, Neoh H min, Masri SN (2023). Epidemiology of multidrug-resistant Klebsiella pneumoniae infection in clinical settings in South-Eastern Asia: a systematic review and meta-analysis. Antimicrob Resist Infect Control [Internet].

[41] Agyepong N, Govinden U, Owusu-Ofori A, Essack SY (2018). Multidrug-resistant gram-negative bacterial infections in a teaching hospital in Ghana. Antimicrob Resist Infect Control [Internet].

[42] Parveen RM, Khan MA, Menezes GA, Harish BN, Parija SC, Hays JP (2011). Extended-spectrum β-lactamase producing Klebsiella pneumoniae from blood cultures in Puducherry, India. Indian J Med Res [Internet].

[43] Obeng-Nkrumah N, Twum-Danso K, Krogfelt KA, Newman MJ (2013). High levels of extended-spectrum beta-lactamases in a major teaching hospital in Ghana: the need for regular monitoring and evaluation of antibiotic resistance. American Journal of Tropical Medicine and Hygiene.

[44] Feglo P, Adu-Sarkodie Y (2016). Antimicrobial resistance patterns of extended spectrum B-lactamase producing Klebsiellae and E. coli isolates from a tertiary hospital in Ghana. Eur Sci J.

[45] Bonnet R (2004). Growing Group of Extended-Spectrum β-Lactamases: the CTX-M Enzymes. Antimicrobial Agents and Chemotherapy.

[46] Mulvey MR, Simor AE (2009). Antimicrobial resistance in hospitals: How concerned should we be?. Canadian Medical Association or its lincensors.

[47] Shoja S, Ansari M, Faridi F, Azad M, Davoodian P, Javadpour S (2018). Identification of Carbapenem-Resistant Klebsiella pneumoniae with Emphasis on New Delhi Metallo-Beta-Lactamase-1 (blaNDM-1) in Bandar Abbas, South of Iran. Microbial Drug Resistance (Larchmont, NY).

[48] Asante KP, Boamah EA, Abdulai MA, Buabeng KO, Mahama E, Dzabeng F (2017). Knowledge of antibiotic resistance and antibiotic prescription practices among prescribers in the Brong Ahafo Region of Ghana; a cross-sectional study. BMC Health Services Research.

[49] Boamah VE, Agyare C, Odoi H, Dalsgaard A (2016). Practices and Factors Influencing the Use of Antibiotics in Selected Poultry Farms in Ghana Antimicrobial Agents. J Antimicrob Agents.

[50] Kimang'a AN (2012). A situational analysis of antimicrobial drug resistance in Africa: are we losing the battle?. Ethiop J Health Sa.

